# Access for Contributors: PLoS Expands Options for Publication of Research and Comment

**DOI:** 10.1371/journal.pmed.0030416

**Published:** 2006-09-26

**Authors:** The PLoS Medicine Editors

Medical researchers—and scientists in general—place a high value on their ability to make independent judgments. Yet the same group often seeks metrics to validate their subjective opinions. One could argue that as rationalists, scientists should have little use for subjectivity, opinions should be supported by verifiable data, and the subjective view of a paper's worth should not be a consideration in editorial decisions about which papers to publish. In reality, however, given the sheer volume of papers published each day, the subjective value of a paper's “worth” is an accepted criterion in scientific publication as no individual could possibly read all papers published in their own field without some guidance as to their likely importance.

So, like other journals presented with many objectively sound submissions, the editors at *PLoS Medicine* have ultimately to make subjective decisions about which papers we accept. As an indication of how far we have come in establishing a “top-tier” open-access medical journal, *PLoS Medicine* now has a rejection rate of greater than 80 percent. We feel that the unwanted side-effect of this rejection rate—and the authors of rejected papers would agree—is that we are turning away many valuable contributions. Some of the papers we reject are clearly not suitable for a general medical journal, but for others that decision is less clear-cut, and most of the submitted manuscripts contain valuable data, even if they sometimes fall short of supporting more ambitious conclusions.

As an organization whose vision is a world of scientific publishing where there is an open-access journal for every paper worth publishing, it is essential that PLoS continues to create additional open-access venues to help reach this goal. Two recent developments take us closer.

*PLoS Clinical Trials* (http://www.plosclinicaltrials.org) was launched five months ago and has already started to broaden the scope of clinical trials reporting by publishing the results of randomized clinical trials from all medical and public health disciplines without regard to the direction of the results, a trial's size, or its “importance.”

Broadening the range of open-access options further, PLoS has recently announced the forthcoming launch of a new and very different publishing project. *PLoS ONE* (http://www.plosone.org) will be an open-access venue for research from every discipline, including all areas of medicine and public health, and will use the capabilities of the Internet to allow readers to participate directly in the publishing process. The growing availability of online tools that allow users to share, filter, link, and annotate online information makes it much easier for readers to find the information that is of most interest to them, and to add value to it. By making use of these advances, we hope that *PLoS ONE* will lead the way towards a new form of scientific discourse that maintains those elements of conventional journals that benefit the scientific and medical community but which also embraces the potential of the Internet to create a more interactive, community-driven literature that invites participation from anyone who has a valuable contribution to make.

*PLoS ONE*We hope that *PLoS ONE* will lead the way towards a new form of scientific discourse.

Papers published by *PLoS ONE* will be held to rigorous standards of scientific quality. Experts will assess whether the results are valid and presented in sufficient detail to allow critical evaluation by readers, and whether the conclusions are fully supported. Speculation is encouraged, but must be clearly marked as such. However, subjective considerations such as “likely impact,” “degree of advance,” or “interest to a general reader” will not play a role in deciding whether an article should be published. Hence, *PLoS ONE* will be able to publish a much wider range of papers than *PLoS Medicine*, for example, can now accommodate. Editorial decisions will be reached rapidly by an extensive editorial board, and accepted articles will be published (in PDF and fully tagged XML formats) in as little as two weeks after acceptance.

And, crucially, published papers will be exposed to post-publication peer review. Readers will have the tools to add comments, questions, related data, and ratings to each article, and authors will be able to update, clarify, and further discuss their findings. *PLoS ONE* will empower researchers and consumers of research to engage in an open discussion on published articles. The goal is to capture the varied and extremely valuable assessment of papers that occurs after publication in a way that is not possible within the limits of conventional Correspondence or Letters sections. It is our hope that such post-publication activity will have many benefits: papers that emerge as influential for a particular field will attract significant annotation and comment, benefiting the authors as well as the readers; discussion will help to link previously unconnected groups or even communities of researchers; free access to this content and interaction will help to level out some of the inequalities between researchers in privileged settings with easy access to information and those who are less well connected. No doubt, there will be some surprises too.

Like the other PLoS journals, *PLoS ONE* will have publication charges. (The PLoS fee waiver policy applies to *PLoS ONE* authors just as for our other journals: if you cannot pay any or part of the publication fee, we will waive or lower it, and editors and reviewers who make decisions about publication have no knowledge of who can pay.) Annotation, on the other hand, carries no charge; all contributions that are deemed valuable will be posted rapidly and are citable.

*PLoS ONE* will be our prototype, but the open-source software that allows this level of online interaction will be added to *PLoS Medicine* shortly after the launch of *PLoS ONE* later this year. What this means is that although we will maintain “subjective” editorial criteria for *PLoS Medicine*, this assessment itself will be open to broader community opinion. We are excited that we will be able to accommodate a much larger number of contributors—authors and annotators—to all PLoS publications and invite you to participate.

